# Leisure activities and disability in activities of daily living among the oldest-old Chinese population: evidence from the Chinese Longitudinal Healthy Longevity Study

**DOI:** 10.18632/aging.103287

**Published:** 2020-06-12

**Authors:** Zhi-Hao Li, Qing Chen, Virginia Byers Kraus, Dong Shen, Xi-Ru Zhang, Pei-Dong Zhang, Wen-Fang Zhong, Yue-Bin Lv, Qing-Mei Huang, Wei-Qi Song, Dong Wang, Xian-Bo Wu, Xiao-Ming Shi, Chen Mao

**Affiliations:** 1 Department of Epidemiology, School of Public Health, Southern Medical University, Guangzhou, Guangdong, China; 2 Duke Molecular Physiology Institute and Division of Rheumatology, Department of Medicine, Duke University School of Medicine, Durham, NC 27701, USA; 3 National Institute of Environmental Health, Chinese Center for Disease Control and Prevention, Beijing, China; 4 School of Health Services Management, Southern Medical University, Guangzhou, Guangdong, China

**Keywords:** leisure activity, disability in activities of daily living, oldest-old, cohort study

## Abstract

Introduction: To investigate the independent and joint effects of leisure activities on disability in activities of daily living (ADL) among the oldest-old Chinese population (aged ≥ 80 years).

Results: A total of 3696 participants with ADL disability were identified during the median follow-up period of 3.1 years. Compared to the participants who “never” watched TV or listened to the radio and who “never” kept domestic animals or pets, those who engaged in these activities “almost every day” had a significantly lower ADL disability risk (adjusted hazard ratios were 0.74 and 0.66, respectively; both *P* < 0.001). Furthermore, participants engaging in multiple leisure activities showed a reduced risk of ADL disability (*P* for trend < 0.001).

Conclusions: Frequently watching TV or listening to the radio and keeping domestic animals or pets was associated with a lower risk of ADL disability among the oldest-old Chinese population.

Methods: We included 12,331 participants (aged ≥ 80 years) (mean [SD] age: 89.5 [7.0] years) who managed to perform ADL independently at baseline in the Chinese Longitudinal Healthy Longevity Survey 1998-2014 waves. Cox proportional hazards models were used to examine whether leisure activities were associated with ADL disability.

## INTRODUCTION

Disability in activities of daily living (ADL), a leading indicator of health status and a major determinant of quality of life, is an increasing major public health concern globally [1–3]. Due to the fact that elderly adults have a high risk of disability, which can create large economic and social burdens [4], it is essential to identify the risk and protective factors for ADL disability, particularly those that are modifiable, to address the challenges posed by rapid societal aging in the coming decades. Leisure activities usually form a relatively large part of people’s daily lives after retirement. The leisure activities of elderly adults have been a common focus of studies investigating health outcomes such as cognitive functioning [5–10], morbidity [11, 12], and mortality [13]. Recently, several studies have found that regular participation in leisure activities may help older adults maintain ADL. For example, previous longitudinal cohort studies [14–16] showed that a higher level of engagement in social activities decreased the risk of incident ADL disability among older adults. Moreover, Nusselder and colleagues [17] reported that participating in physical activities in midlife was associated with a lower risk and later onset of disability in late life, and the types of leisure activities may vary by country [14–17]. However, existing studies have mostly focused on developed countries, and evidence regarding the potential roles of specific types of leisure activities on ADL disability is limited.

The oldest-old population (aged 80 or older) has a high incidence of ADL disability [18] and is the fastest growing subgroup of the older population [19]. However, most studies on the association between leisure activities and ADL disability have focused on relatively younger elderly adults, and studies concerning the association on the oldest-old adults are scarce. In this context, the limited representation of the oldest-old individuals in previous studies constitutes another knowledge gap [20].

We therefore conducted a large community-based longitudinal study on oldest-old individuals (aged ≥ 80 years) in China with the aim of investigating the associations between leisure activities and the risk of ADL disability, examining each activity separately and in combination.

## RESULTS

### Baseline characteristics

Table 1 shows the characteristics of the participants at baseline. Among the 12,331 participants (mean [SD] age: 89.5 [7.0] years), 6946 (56.3%) were women. Compared to participants with ADL independence, those who developed ADL disability were relatively likely to be women (63.5% vs 53.2%), to live in urban areas (44.3% vs 38.1%), to live with others (86.4% vs 82.2%), to be not married (80.1% vs 75.4%), and to be illiterate (68.7% vs 64.4%) (all *P* < 0.001). Moreover, participants with ADL disability tended to have a greater prevalence of stroke, heart disease, depression symptoms and cognitive impairment (all *P* < 0.05).

**Table 1:** Baseline characteristics of participants stratified by the development of disability in activities of daily living.

**Characteristics**	**Total (*n* =12,331)**	**No. (%) ADL independence (*n* = 8635)**	**No. (%) ADL disability (*n* = 3696)**	***P***
Age, mean (SD), years	89.52 (7.0)	89.00 (6.9)	90.73 (7.2)	< 0.001
Women	6946 (56.3)	4598 (53.2)	2348 (63.5)	< 0.001
Residence				< 0.001
Urban	4923 (39.9)	3287 (38.1)	1636 (44.3)	
Rural	7408 (60.1)	5348 (61.9)	2060 (55.7)	
Co-residence status				< 0.001
Living alone	2039 (16.5)	1535 (17.8)	504 (13.6)	
Living with others	10,292 (83.5)	7100 (82.2)	3192 (86.4)	
Marital status				< 0.001
Married	2858 (23.2)	2124 (24.6)	734 (19.9)	
Not married	9473 (76.8)	6511 (75.4)	2962 (80.1)	
Educational level, years				< 0.001
0	8102 (65.7)	5562 (64.4)	2540 (68.7)	
≥ 1	4229 (34.3)	3073 (35.6)	1156 (31.3)	
BMI, kg/m^2^				0.015
< 18.5	5491 (44.5)	3892 (45.1)	1599 (43.3)	
18.5-23.9	5292 (42.9)	3705 (42.9)	1587 (42.9)	
≥ 24.0	1548 (12.6)	1038 (12.0)	510 (13.8)	
Smoking status				< 0.001
Current smoker	2293 (18.6)	1692 (19.6)	601 (16.3)	
Former smoker	1685 (13.7)	1215 (14.1)	470 (12.7)	
Nonsmoker	8353 (67.7)	5728 (66.3)	2625 (71.0)	
Alcohol status				< 0.001
Current drinker	2775 (22.5)	2029 (23.5)	746 (20.2)	
Former drinker	1166 (9.5)	838 (9.7)	328 (8.9)	
Nondrinker	8390 (68.0)	5768 (66.8)	2622 (70.9)	
Frequent fresh vegetable consumption	4140 (33.6)	2919 (33.8)	1221 (33.0)	0.420
Frequent fresh fruit consumption	10,893 (88.3)	7726 (89.5)	3167 (85.7)	< 0.001
Physical exercise	6613 (53. 7)	4724 (54.7)	1889 (51.1)	< 0.001
Hypertension	5087 (41.2)	3605 (41.7)	1482 (40.1)	0.092
Diabetes mellitus	119 (1.0)	77 (0.9)	42 (1.1)	0.241
Respiratory disease	1252 (10.1)	890 (10.3)	362 (9.8)	0.406
Stroke	294 (2.4)	185 (2.1)	109 (2.9)	0.009
Heart disease	759 (6.2)	496 (5.7)	263 (7.1)	0.004
Depression symptoms	510 (4.5)	291 (3.4)	219 (5.9)	< 0.001
Cognitive impairment	1677 (13.7)	1097 (12.7)	580 (15.7)	< 0.001

ADL: activities of daily living; SD: standard deviation; BMI: body mass index. Data are presented as *n* (percent) unless otherwise indicated.

### Associations between leisure activities and ADL disability

During a median follow-up period of 3.1 years (75,737 person-years), 3696 participants with ADL disability were identified. Table 2 presents leisure activities and their associations with the risk of ADL disability. In the fully adjusted model (model 2), compared with “never” watching TV or listening to the radio, engaging in these activities “almost every day” (HR: 0.74, 95% CI: 0.68-0.81, *P* < 0.001) was more strongly associated with a reduced risk of ADL disability than engaging in them “sometimes” (HR: 0.80, 95% CI: 0.73-0.87, *P* < 0.001). Additionally, caring for animals “almost every day” (HR: 0.66, 95% CI: 0.59-0.73, *P* < 0.001) was more strongly associated with a lower risk of ADL disability than caring for them “sometimes” (HR: 0.75, 95% CI: 0.67-0.84, *P* < 0.001). However, there were no significant associations between the risk of ADL disability and the frequency of reading books or newspapers, playing cards or mahjong, gardening, or attending religious activities, with the exception of reading books or newspapers “almost every day” (HR: 0.86, 95% CI: 0.75-0.99, *P* = 0.035), playing cards or mahjong “sometimes” (HR: 0.86, 95% CI: 0.76-0.97, *P* = 0.014), and participating in gardening “almost every day” (HR: 0.85, 95% CI: 0.74-0.97, *P* = 0.018). An examination of the effects of participating in a variety of leisure activities on ADL disability showed that engaging in multiple leisure activities was associated with a decreased risk of ADL disability (*P* for trend < 0.001).

**Table 2:** Associations between leisure activities and disability in activities of daily living.

**Leisure activity**	**n/N**	**Model 1^a^**		**Model 2^b^**	
		**HR (95% CI)**	***P***	**HR (95% CI)**	***P***
Watching TV or listening to the radio					
Never	1527/4540	1.00 (reference)	-	1.00 (reference)	-
Sometimes	938/3295	0.81 (0.74-0.88)	< 0.001	0.80 (0.73-0.87)	< 0.001
Almost every day	1231/4496	0.81 (0.75-0.87)	< 0.001	0.74 (0.68-0.81)	< 0.001
Reading books or newspapers					
Never	3120/10,202	1.00 (reference)	-	1.00 (reference)	-
Sometimes	244/933	0.92 (0.80-1.05)	0.202	0.88 (0.76-1.02)	0.096
Almost every day	332/1196	1.02 (0.91-1.15)	0.715	0.86 (0.75-0.99)	0.035
Keeping domestic animals or pets					
Never	2940/9126	1.00 (reference)	-	1.00 (reference)	-
Sometimes	355/1392	0.69 (0.61-0.77)	< 0.001	0.75 (0.67-0.84)	< 0.001
Almost every day	401/1813	0.57 (0.51-0.63)	< 0.001	0.66 (0.59-0.73)	< 0.001
Playing cards or mahjong					
Never	3210/10,456	1.00 (reference)	-	1.00 (reference)	-
Sometimes	318/1228	0.86 (0.76-0.97)	0.011	0.86 (0.76-0.97)	0.014
Almost every day	168/647	0.90 (0.77-1.05)	0.166	0.86 (0.73-1.01)	0.628
Gardening					
Never	3232/10,722	1.00 (reference)	-	1.00 (reference)	-
Sometimes	212/725	1.00 (0.87-1.15)	0.971	1.02 (0.88-1.18)	0.812
Almost every day	252/884	0.89 (0.78-1.01)	0.069	0.85 (0.74-0.97)	0.018
Attending religious activities					
Never	3209/10,549	1.00 (reference)	-	1.00 (reference)	-
Sometimes	361/1368	0.91 (0.82-1.01)	0.089	0.93 (0.83-1.04)	0.194
Almost every day	126/414	0.96 (0.81-1.15)	0.692	0.94 (0.79-1.13)	0.507
Number of leisure activities					
0	1078/2875	1.00 (reference)	-	1.00 (reference)	-
1	1140/3859	0.76 (0.70-0.83)	< 0.001	0.72 (0.66-0.79)	< 0.001
2	877/3233	0.69 (0.63-0.76)	< 0.001	0.63 (0.57-0.69)	< 0.001
3	415/1606	0.62 (0.55-0.70)	< 0.001	0.50 (0.44-0.57)	< 0.001
4+	186/755	0.59 (0.50-0.69)	< 0.001	0.46 (0.39-0.54)	< 0.001
*P* for trend			< 0.001		< 0.001

HR: hazard ratio; CI: confidence interval;

^a^ Model 1: adjusted for age and gender;

^b^ Model 2: further adjusted for residence, co-residence status, marital status, educational level, body mass index, smoking status, alcohol status, frequent fresh vegetable consumption, frequent fresh fruit consumption, physical exercise, hypertension, diabetes mellitus, respiratory disease, stroke, heart disease, depression symptoms, cognitive impairment, and participation in other leisure activities

### Subgroup analysis

We performed a subgroup analysis stratified by gender using the fully adjusted model (Table 3). Attending religious activities “almost every day” or “sometimes” was significantly associated with a lower risk of ADL disability among men (all *P* < 0.05) but not among women (all *P* > 0.05) (*P* for interaction = 0.014), and there were no significant effects on the interactions of associations between other leisure activities and ADL disability stratified by gender (all *P* for interaction > 0.05). Moreover, we conducted a stratified analysis by age group (< 90 and ≥ 90 years) using fully adjusted models (Table 4). Attending religious activities “sometimes” was associated with a reduced risk of ADL disability among participants aged 80-89 years (*P* = 0.003) but not among participants aged 90 years or older (*P* = 0.239) (*P* for interaction = 0.013), and there were no significant effects on the interactions of associations between other leisure activities and ADL disability stratified by age (all *P* for interaction > 0.05).

**Table 3:** Associations between leisure activities and disability in activities of daily living in groups stratified by gender.

**Leisure activity**	**Men (*n* = 5385)**			**Women (*n* = 6946)**			***P* for interaction**
	**n/N**	**HR (95% CI) ^a^**	***P***	**n/N**	**HR (95% CI)**	***P***	
Watching TV or listening to the radio							0.768
Never	1088/1491	1.00 (reference)	-	1124/3049	1.00 (reference)	-	
Sometimes	337/1416	0.83 (0.71-0.96)	< 0.001	601/1879	0.78 (0.71-0.87)	< 0.001	
Almost every day	608/2478	0.81 (0.70-0.94)	< 0.001	623/2018	0.70 (0.63-0.78)	< 0.001	
Reading books or newspapers							0.438
Never	896/3619	1.00 (reference)	-	2224/6583	1.00 (reference)	-	
Sometimes	175/740	0.86 (0.72-1.02)	0.389	69/193	1.00 (0.77-1.29)	0.988	
Almost every day	277/1026	0.87 (0.74-1.03)	0.048	55/170	0.81 (0.60-1.09)	0.167	
Keeping domestic animals or pets							
Never	1093/4024	1.00 (reference)	-	2126/6269	1.00 (reference)	-	0.961
Sometimes	130/620	0.79 (0.66-0.96)	0.007	109/341	0.73 (0.63-0.84)	< 0.001	
Almost every day	125/741	0.68 (0.56-0.83)	< 0.001	113/336	0.64 (0.56-0.84)	< 0.001	
Playing cards or mahjong							0.095
Never	1103/4246	1.00 (reference)	-	2107/6210	1.00 (reference)	-	
Sometimes	161/764	0.79 (0.67-0.93)	0.006	157/464	0.95 (0.80-1.12)	0.631	
Almost every day	84/375	0.81 (0.64-1.02)	0.071	84/272	0.92 (0.82-1.16)	0.491	
Gardening							0.961
Never	1106/4453	1.00 (reference)	-	1847/5102	1.00 (reference)	-	
Sometimes	103/384	1.10 (0.89-1.36)	0.389	225/772	0.95 (0.78-1.16)	0.630	
Almost every day	139/548	0.82 (0.68-1.00)	0.048	276/1072	0.86 (0.70-1.05)	0.134	
Attending religious activities							0.014
Never	1197/4696	1.00 (reference)	-	2012/5853	1.00 (reference)	-	
Sometimes	120/545	0.80 (0.66-0.97)	0.025	241/823	0.99 (0.86-1.14)	0.913	
Almost every day	31/144	0.63 (0.44-0.90)	0.014	318/441	1.11 (0.90-1.37)	0.321	
Number of leisure activities							0.720
0	1078/2875	1.00 (reference)	-	1078/2875	1.00 (reference)	-	
1	1140/3859	0.71 (0.61-0.83)	< 0.001	1140/3859	0.72 (0.65-0.80)	< 0.001	
2	877/3233	0.60 (0.51-0.71)	< 0.001	877/3233	0.63 (0.56-0.71)	< 0.001	
3	415/1606	0.48 (0.40-0.58)	< 0.001	415/1606	0.51 (0.44-0.61)	< 0.001	
4+	186/755	0.44 (0.35-0.56)	< 0.001	186/755	0.46 (0.35-0.60)	< 0.001	
*P* for trend			< 0.001			< 0.001	

HR: hazard ratio; CI: confidence interval;

^a^ Adjusted for age, residence, co-residence status, marital status, educational level, body mass index, smoking status, alcohol status, frequent fresh vegetable consumption, frequent fresh fruit consumption, physical exercise, hypertension, diabetes mellitus, respiratory disease, stroke, heart disease, depression symptoms, cognitive impairment, and participation in other leisure activities.

**Table 4:** Associations between leisure activities and disability in activities of daily living in groups stratified by age.

**Leisure activity**	**< 90 years (*n* = 6552)**			**≥ 90 years (*n* = 5779)**			***P* for interaction**
	**n/N**	**HR (95% CI) ^a^**	***P***	**n/N**	**HR (95% CI)**	***P***	
Watching TV or listening to the radio							0.586
Never	533/1884	1.00 (reference)	-	994/2656	1.00 (reference)	-	
Sometimes	455/1804	0.81 (0.71-0.92)	0.001	483/1491	0.78 (0.70-0.87)	< 0.001	
Almost every day	686/2864	0.74 (0.65-0.84)	< 0.001	545/1087	0.75 (0.66-0.84)	< 0.001	
Reading books or newspapers							0.371
Never	1321/5108	1.00 (reference)	-	1799/5094	1.00 (reference)	-	
Sometimes	141/611	0.83 (0.68-1.00)	0.054	103/322	0.97 (0.78-1.21)	0.798	
Almost every day	212/833	0.86 (0.72-1.04)	0.124	120/363	0.88 (0.71-1.10)	0.253	
Keeping domestic animals or pets							0.938
Never	1267/4594	1.00 (reference)	-	1834/5244	1.00 (reference)	-	
Sometimes	175/769	0.76 (0.65-0.89)	< 0.001	88/268	0.74 (0.63-0.87)	< 0.001	
Almost every day	232/1189	0.66 (0.57-0.76)	< 0.001	100/267	0.68 (0.58-0.80)	< 0.001	
Playing cards or mahjong							0.759
Never	1382/5280	1.00 (reference)	-	1828/5176	1.00 (reference)	-	
Sometimes	184/815	0.86 (0.73-1.01)	0.058	134/413	0.87 (0.72-1.04)	0.122	
Almost every day	108/457	0.92 (0.75-1.13)	0.425	60/190	0.81 (0.62-1.06)	0.121	
Gardening							0.748
Never	1398/5478	1.00 (reference)	-	1673/4532	1.00 (reference)	-	
Sometimes	124/457	1.08 (0.89-1.31)	0.439	180/623	0.96 (0.77-1.19)	0.688	
Almost every day	152/617	0.88 (0.73-1.05)	0.154	169/624	0.79 (0.63-0.98)	0.029	
Attending religious activities							0.013
Never	1439/5494	1.00 (reference)	-	1770/5055	1.00 (reference)	-	
Sometimes	179/836	0.79 (0.67-0.92)	0.003	182/532	1.10 (0.94-1.29)	0.239	
Almost every day	56/222	0.90 (0.69-1.18)	0.455	70/192	0.98 (0.77-1.25)	0.862	
Number of leisure activities							0.141
0	334/1022	1.00 (reference)	-	744/1831	1.00 (reference)	-	
1	487/1907	0.67 (0.58-0.77)	< 0.001	653/1952	0.75 (0.68-0.84)	< 0.001	
2	490/1991	0.60 (0.52-0.69)	< 0.001	387/1242	0.63 (0.56-0.72)	< 0.001	
3	250/1099	0.45 (0.38-0.54)	< 0.001	165/510	0.57 (0.47-0.68)	< 0.001	
4+	113/533	0.43 (0.34-0.53)	< 0.001	73/222	0.49 (0.38-0.64)	< 0.001	
*P* for trend			< 0.001			< 0.001	

HR: hazard ratio; CI: confidence interval;

^a^Adjusted for gender, residence, co-residence status, marital status, educational level, body mass index, smoking status, alcohol status, frequent fresh vegetable consumption, frequent fresh fruit consumption, physical exercise, hypertension, diabetes mellitus, respiratory disease, stroke, heart disease, depression symptoms, cognitive impairment, and participation in other leisure activities.

### Sensitivity analysis

Our sensitivity analyses did not find substantial changes in the results after we adjusted for the time of recruitment (Supplementary Table 2), excluded participants with missing covariate data (Supplementary Table 3), and limited the analyses to participants with at least 2 years of follow-up data (Supplementary Table 4).

## DISCUSSION

In the large population-based prospective longitudinal cohort study of 12,331 Chinese oldest-old individuals, we investigated the associations between leisure activities and ADL disability. Our analyses revealed that frequently watching TV or listening to the radio and keeping domestic animals or pets was associated with a lower risk of ADL disability, and the associations were independent of the covariates (such as sociodemographic information, lifestyle behaviors and prevalence of chronic diseases). Moreover, participation in an increasing number of leisure activities was associated with a significantly decreased risk of ADL disability. Previous studies have reported that frequently prolonged TV watching could lead to unhealthy outcomes such as obesity [21], diabetes [22] and cardiovascular disease [22, 23], which may influence the risk of ADL disability. However, our study found that more frequently watching TV or listening to the radio was beneficial for maintaining normal ADL. The explanation for this finding might be that most of the oldest-old Chinese individuals in our study were illiterate and thus had difficulty reading books or newspapers and lacked other sources of information. Meanwhile, watching TV or listening to the radio is not only intellectually challenging and informative, but also an easier leisure activity allowing individuals to relax and obtain information from the outside world [13]. Moreover, previous studies have found that frequently watching TV might be more likely to produce positive emotions of being engaged in life and enhance life satisfaction [24] and happiness [25], which may help elderly adults perform ADL independently. Unfortunately, the present study did not obtain information on the level of happiness or satisfaction or the type of TV or radio programs that participants watched.

Furthermore, our study found that keeping domestic animals or pets was associated with a decreased risk of ADL disability. In line with this finding, previous studies have reported that keeping pets can not only improve quality of life [26] but also promote physical health [27, 28]. Keeping pets has been shown to be beneficial for maintaining or slightly enhancing ADL performance in older adults [29]. For example, a study from Canada including more than 1000 older adults [30] suggested that the loss of the ability to perform ADL progressed at a greater rate for persons who did not own pets than for pet owners. Several studies have found that dog walking may encourage elderly individuals to engage in beneficial physical activities and to preserve their functionality [31]; additionally, cat ownership might alleviate negative moods, with the observed effect being comparable to the effect of a human partner [32]. Furthermore, animal-assisted therapy has been widely used as a therapeutic tool for various psychiatric populations; this treatment was found to reinforce the ability to perform ADL, including personal hygiene and independent self-care, by using cats and dogs as “modeling companions” [33]. This finding implies that maintaining ADL by keeping domestic animals or pets, especially for the oldest-old population, might be important for preventing ADL disability.

Interestingly, our study also revealed that compared to older women, the oldest-old men were associated with a lower predicted probability of having ADL disability in the context of attending religious activities. Idler and colleagues [34] found that attending public religious activities could have significant protective effects against ADL disability among men. Moreover, longitudinal studies [35, 36] showed that older men who were more active in religious pursuits experienced greater longevity after controlling for covariates. However, according to a large prospective long-term cohort study [37], frequently participation in religious activities was associated with a significantly lower risk of all-cause mortality among US women. Religious activity involvement, meaning active participation in the social life of the church, was associated with a strong positive effect on the ADL independence of the elderly [34]. Possibly, the difference in religious culture and the small sample size of older men participating in religious activities led to this gender difference in our study.

Meanwhile, the present study found that the risk of ADL disability progressively decreased with participation in an increasing number of leisure activities. Similarly, a previous cohort study indicated that older adults who participated in more leisure activities were less likely to develop ADL disability than those who engaged in fewer activities [38, 39]. Therefore, encouraging the oldest-old population to engage in a greater number of leisure activities could be beneficial to reduce the risk of ADL disability.

The main strengths of this study included the large sample size of the oldest-old Chinese population, the ability to control for various known and potential confounding factors, and the use of community-based prospective data. Nevertheless, our study had several limitations. First, this study was limited by its observational design; therefore, a causal relationship cannot be concluded. Second, although we controlled for many potential and previously described confounders to optimize the outcome of this study, including health problems (i.e., chronic conditions and depressive symptoms) and physical resources (i.e., body mass index and blood pressure), other potential confounding factors (i.e., waist circumference and lung function) were not measured or controlled in this study, which might be one of the driving factors underlying the associations of leisure activities with ADL disability. Third, because participants lost-to-follow-up at the first follow-up survey were excluded, this might have caused potential selection bias that affected our findings. Moreover, our study investigated a finite number of leisure activities; hence, the number of activities evaluated was not consistent across different activity types (e.g., mental, social and physical activities). In addition, the time to ADL disability was defined as the period from baseline to the first time a disability was developed; however, the participants might have developed ADL disability at any time during a 2- to 3-year interval. Therefore, it was impossible to determine the exact date of the event, which may have biased the findings.

In conclusion, later-life frequently participation in leisure activities, including watching TV or listening to the radio and keeping domestic animals or pets, was associated with a lower risk of ADL disability among the oldest-old Chinese population. Our results suggest that encouraging the incorporation of various leisure activities at advanced ages may enhance quality of life, probably by improving the ability to perform ADL independently. This finding has important public health implications, as encouraging elderly adults to participate regularly in multiple (and potentially modifiable) leisure activities may help prevent the onset of ADL disability. Future studies are needed to examine the associations between leisure activity, especially watching TV and keeping pets, and different levels of disability or the number of items individuals are incapable of performing.

## MATERIALS AND METHODS

### Study design and participants

**Figure 1:**
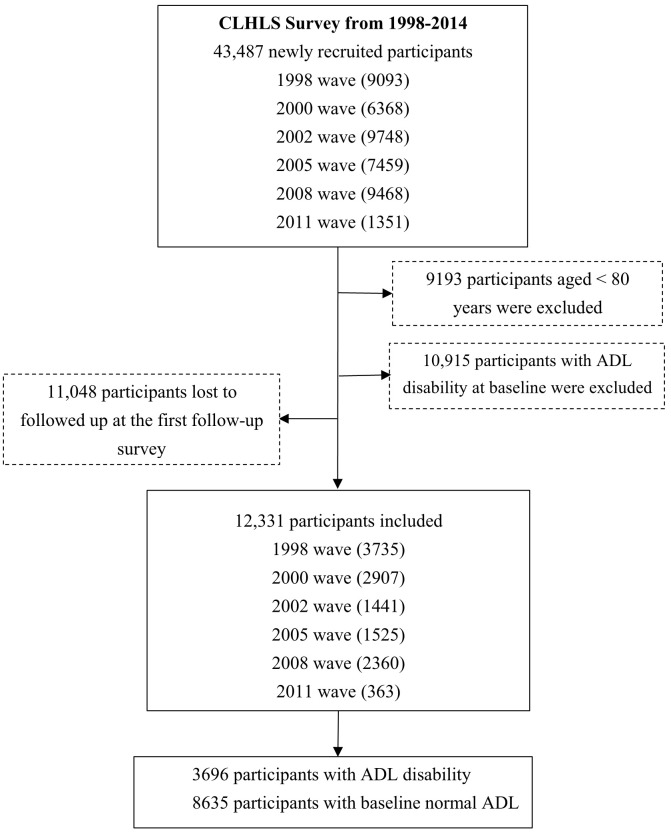
**Flowchart of participant enrollment.**

The study was derived from the Chinese Longitudinal Healthy Longevity Survey (CLHLS), which is a national longitudinal survey on determinants of healthy aging. Details of the survey design and participants have been described previously [1, 40]. Briefly, the CLHLS is the largest study of its kind and includes the largest sample of the oldest-old Chinese population aged 80 or older [1, 41]. Studies have shown that the Chinese version of the questionnaire yields reliable and effective responses [42–44]. The CLHLS was conducted with a randomly selected sample of elderly adults from 23 provinces in China; the sampling frame accounted for approximately 85% of the total population of China and covered 806 counties and districts. The survey was first launched in 1998, and follow-up surveys and recruitment of new participants were performed in 2000, 2002, 2005, 2008, 2011 and 2014. All centenarians (age 100+ years) in selected counties or cities were interviewed together with one nearby octogenarian (age 80-89 years) and nonagenarian (age 90-99 years) matched in terms of geographical unit and gender [10]. Among the 43,487 participants in the CLHLS, those excluded consisted of 9193 participants aged < 80 years, 10,915 participants with ADL disability at baseline, and 11,048 participants lost to follow-up at the first follow-up survey (Figure 1). The final sample included 12,331 participants: 5385 men and 6946 women. We compared the results for the included participants to those from datasets excluding participants who were not followed-up and found that the distributions of variables from the participants included were similar to those obtained when excluding participants who were not followed-up in the first survey or died (Supplementary Table 1). The CLHLS study was approved by the Research Ethics Committee of Peking University (IRB00001052-13074). All participants or their proxy participants signed written informed consent forms.

### Measurement of leisure activities

Data on self-reported leisure activities were collected through in-home interviews. At baseline, the participants were interviewed about their participation in leisure activities, including watching TV or listening to the radio, reading books or newspapers, keeping domestic animals or pets, playing cards or mahjong, gardening, and attending religious activities. The frequency of participation in each activity was described as “almost every day”, “sometimes”, or “never”. “Almost every day” was defined as participating in leisure activities at least 5 times per week, whereas “sometimes” was defined as participation at least once a week, once a month or once per quarter rather than almost every day. The binary variable of participation in leisure activities was coded as “1” if the answer was “almost every day” or “sometimes” and as “0” if the answer was “never” for each leisure activity. We then summed the scores for the six leisure activities, and the results were classified as 0, 1, 2, 3, and 4+ [13].

### Measurement of ADL disability

ADL was measured based on participants’ self-reported results. ADL disability was defined as participants who could not perform or needed help at least one of six basic self-care tasks — feeding, dressing, transferring, bathing, toileting and continence — according to the Katz index scale [41, 44, 45]. Each ADL item was scored 1 or 0, where 1 indicated the ability to perform the activity independently and 0 indicated the inability to perform the activity independently. The time to the first known ADL disability was the length of time (months) for the survival analysis. For participants who did not develop ADL disability, the censoring time (months) for statistical analysis was the time of the last assessment.

### Covariates

Data on the independent variables were collected and classified as follows: sociodemographic information, including age (years), gender (men or women), residence (urban or rural), co-residence status (living alone or living with others), marital status (married or not married), educational level (years, 0 or ≥ 1), and body mass index (BMI < 18.5, 18.5-23.9, or ≥ 24.0 kg/m^2^); lifestyle behaviors, including smoking status (current smoker, former smoker, or nonsmoker), alcohol status (current drinker, former drinker, or nondrinker), frequent fresh fruit consumption (yes or no), frequent fresh vegetable consumption (yes or no), and physical exercise (yes or no); and prevalence of hypertension (yes or no), diabetes mellitus (yes or no), respiratory disease (yes or no), stroke (yes or no), heart disease (yes or no), depressive symptoms (yes or no) and cognitive impairment (yes or no). Depressive symptoms were measured with two self-assessment questions: i) Have you ever been sad, blue or depressed for 2 weeks or more? ii) Have you lost interest in things, such as hobbies, work, or activities that usually give you pleasure? Participants who had at least one positive answer were defined as having depressive symptoms [10]. Cognitive impairment was assessed by the Chinese version of the Mini-Mental State Examination (MMSE), whose score ranges from 0-30; a score < 18 was defined as indicating cognitive impairment [46–48]. All covariate information was obtained using a standardized and structured questionnaire in the baseline survey [13].

### Statistical analysis

Overall, less than 3% of the data for the covariates at baseline was missing, and a multiple imputation method was used to correct for missing values and reduce the potential inferential bias [18]. For participants who remained free of ADL disability at their final survey point, the follow-up time was calculated from the baseline survey to the final survey. For participants who developed ADL disability, the onset time of ADL disability was assumed to be at the middle point of the two rounds of surveys due to its occult onset [44, 49]; therefore, person-years of follow-up were defined as the full time during which the participants remained free of ADL disability plus half of the follow-up time during which ADL disability developed. Means and standard deviations (SD) (continuous variables) or numbers and percentages (categorical variables) were used to describe the participants’ characteristics at baseline. We used the *t*-test or *χ^2^* test to compare the baseline characteristics of the participants who did or did not experience ADL disability at follow-up.

Cox proportional hazards models were applied to estimate the hazard ratios (HRs) and 95% confidence intervals (95% CIs) for ADL disability associated with baseline specific leisure activities over the follow-up period. Two sets of models were adopted. The basic model (model 1) was adjusted for baseline age and gender. The fully adjusted model (model 2) was adjusted for additional variables, including educational level, marital status, residence status, co-residence status, BMI, smoking status, alcohol status, frequent fresh vegetable consumption, frequent fresh fruit consumption, physical exercise, hypertension, diabetes mellitus, respiratory disease, stroke, heart disease, depression symptoms, cognitive impairment, and participation in other leisure activities. The Cox proportional hazards assumption was performed with Schoenfeld residual plots [50]; we found no evidence of a violation of the assumption in the present study. We examined the associations between participation in a certain number of leisure activities (0, 1, 2, 3 and 4+) and ADL disability in the fully adjusted model.

Subgroup analyses were performed according to gender (men and women) and age (< 90 and ≥ 90 years) using the fully adjusted model. A likelihood ratio test was conducted to test for interaction. Furthermore, we conducted several sensitivity analyses to determine the robustness of our primary results by i) adjusting for the year of recruitment, ii) restricting analyses to participants without missing covariate data, and iii) excluding all participants who developed ADL disability during the first two years of follow-up. The analyses were performed using R software, version 3.6.0 (R Development Core Team, Vienna, Austria). The results were deemed statistically significant at *P* < 0.05 (two-tailed) for all analyses. Since multiple interactions were tested, the Bonferroni correction was used to correct multiple testing conservatively, and a significance level of 0.05/6 = 0.008 was set.

## Supplementary Material

Supplementary Tables
