# Supplementary material for: Leisure activities and disability in activities of daily living among the oldest-old Chinese population: evidence from the Chinese Longitudinal Healthy Longevity Study
Source: Aging (Albany NY). 2020 Jun 12;12(11):10687–703. doi: 10.18632/aging.103287 (PMC7346052; doi:10.18632/aging.103287)
Supplement: Supplementary Tables [file aging-12-103287-s001..pdf]

SUPPLEMENTARY TABLES

Supplementary Table 1. Distributions of variables of included participants compared to results from datasets excluding participants due to lack of follow-up.

Characteristics	Participants included (n =12,331)	Participants excluded due to lack of follow-up (n =11,048)
Age, mean (SD), years	89.52 (7.0)	92.93 (7.2)
Women	6946 (56.3)	6332 (57.3)
Residence		
Urban	4923 (39.9)	4298 (38.9)
Rural	7408 (60.1)	6750 (61.1)
Co-residence status		
Living alone	2039 (16.5)	1863 (16.9)
Living with others	10,292 (83.5)	9185 (83.1)
Marital status		
Married	2858 (23.2)	1690 (15.3)
Not married	9473 (76.8)	9358 (84.7)
Educational level, years		
0	8102 (65.7)	7676 (69.9)
≥ 1	4229 (34.3)	3313 (30.1)
BMI, kg/m^2		
< 18.5	5491 (44.5)	4699 (51.1)
18.5-23.9	5292 (42.9)	3659 (39.8)
≥ 24.0	1548 (12.6)	840 (9.1)
Smoking status		
Current smoker	2293 (18.6)	1912 (17.3)
Former smoker	1685 (13.7)	1534 (13.9)
Nonsmoker	8353 (67.7)	7595 (68.8)
Alcohol status		
Current drinker	2775 (22.5)	2265 (20.5)
Former drinker	1166 (9.5)	1163 (10.5)
Nondrinker	8390 (68.0)	7610 (68.9)
Frequent fresh vegetable consumption	4140 (33.6)	4352 (39.4)
Frequent fresh fruit consumption	10,893 (88.3)	9831 (89.0)
Physical exercise	6613 (53.7)	6888 (62.4)
Depression symptoms	510 (4.5)	795 (7.2)
Cognitive impairment	1677 (13.7)	2191 (22.5)
Hypertension	5087 (41.2)	4832 (45.8)
Diabetes mellitus	119 (1.0)	130 (1.2)
Respiratory disease	1252 (10.1)	1225 (11.1)
Stroke	294 (2.4)	264 (2.4)
Heart disease	759 (6.2)	666 (6.1)

ADL: activities of daily living; SD: standard deviation; BMI: body mass index. Data are presented as n (percent) unless otherwise indicated.

Supplementary Table 2. Association between leisure activities and disability in activities of daily living additionally adjusted for time of recruitment.

Leisure activity	Disability in activities of daily living	
	HR (95% CI) [a]	*P*
Watching TV or listening to the radio		
Never	1.00 (reference)	-
Sometimes	0.82 (0.75-0.89)	< 0.001
Almost every day	0.82 (0.75-0.90)	< 0.001
Reading books or newspapers		
Never	1.00 (reference)	-
Sometimes	0.88 (0.76-1.01)	0.072
Almost every day	0.83 (0.72-0.96)	0.014
Gardening		
Never	1.00 (reference)	-
Sometimes	0.71 (0.64-0.80)	< 0.001
Almost every day	0.66 (0.60-0.74)	< 0.001
Playing cards or mah-jong		
Never	1.00 (reference)	-
Sometimes	0.89 (0.79-1.01)	0.672
Almost every day	0.91 (0.77-1.07)	0.236
Keeping domestic animals or pets		
Never	1.00 (reference)	-
Sometimes	1.00 (0.87-1.16)	0.972
Almost every day	0.86 (0.75-0.98)	0.293
Attending religious activities		
Never	1.00 (reference)	-
Sometimes	0.90 (0.80-1.00)	0.051
Almost every day	0.90 (0.77-1.07)	0.236
Number of leisure activities		
0	1.00 (reference)	-
1	0.72 (0.66-0.79)	< 0.001
2	0.63 (0.57-0.69)	< 0.001
3	0.50 (0.44-0.57)	< 0.001
4+	0.46 (0.39-0.54)	< 0.001
P for trend		< 0.001

HR: hazard ratio; CI: confidence interval

[a] Adjusted for age and sex, residence, co-residence status, marital status, educational level, body mass index, smoking status, alcohol status, frequent fresh vegetable consumption, frequent fresh fruit consumption, physical exercise, hypertension, diabetes mellitus, respiratory disease, stroke, heart disease, depression symptoms, cognitive impairment, and participation in other leisure activities.

Supplementary Table 3. Association between leisure activities and disability in activities of daily living excluding participants with missing data for the covariates.

Leisure activity	Disability in activities of daily living	
	HR (95% CI) [a]	*P*
Watching TV or listening to the radio		
Never	1.00 (reference)	-
Sometimes	0.80 (0.73-0.87)	< 0.001
Almost every day	0.74 (0.68-0.81)	< 0.001
Reading books or newspapers		
Never	1.00 (reference)	-
Sometimes	0.89 (0.77-1.04)	0.141
Almost every day	0.86 (0.75-1.00)	0.049
Gardening		
Never	1.00 (reference)	-
Sometimes	0.75 (0.67-0.84)	< 0.001
Almost every day	0.64 (0.58-0.72)	< 0.001
Playing cards or mah-jong		
Never	1.00 (reference)	-
Sometimes	0.85 (0.75-0.96)	0.010
Almost every day	0.85 (0.72-1.00)	0.046
Keeping domestic animals or pets		
Never	1.00 (reference)	-
Sometimes	1.05 (0.90-1.21)	0.538
Almost every day	0.85 (0.73-0.98)	0.141
Attending religious activities		
Never	1.00 (reference)	-
Sometimes	0.94 (0.84-1.05)	0.293
Almost every day	0.89 (0.74-1.08)	0.235
Number of leisure activities		
0	1.00 (reference)	-
1	0.72 (0.66-0.79)	< 0.001
2	0.63 (0.57-0.69)	< 0.001
3	0.50 (0.44-0.57)	< 0.001
4+	0.46 (0.39-0.54)	< 0.001
P for trend		< 0.001

HR: hazard ratio; CI: confidence interval

[a] Adjusted for age and sex, residence, co-residence status, marital status, educational level, body mass index, smoking status, alcohol status, frequent fresh vegetables consumption, frequent fresh fruit consumption, physical exercise, hypertension, diabetes mellitus, respiratory disease, stroke, heart disease, depression symptoms, cognitive impairment, and participation in other leisure activities.

Supplementary Table 4. Association between leisure activities and disability in activities of daily living excluding participants who lost to follow-up at least two years.

Leisure activity	Disability in activities of daily living	
	HR (95% CI) [a]	*P*
Watching TV or listening to the radio		
Never	1.00 (reference)	-
Sometimes	0.74 (0.68-0.82)	< 0.001
Almost every day	0.72 (0.65-0.79)	< 0.001
Reading books or newspapers		
Never	1.00 (reference)	-
Sometimes	0.94 (0.80-1.11)	0.444
Almost every day	0.90 (0.77-1.06)	0.211
Gardening		
Never	1.00 (reference)	-
Sometimes	0.67 (0.59-0.76))	< 0.001
Almost every day	0.59 (0.53-0.67)	< 0.001
Playing cards or mah-jong		
Never	1.00 (reference)	-
Sometimes	0.85 (0.74-0.97)	0.016
Almost every day	0.82 (0.68-0.98)	0.033
Keeping domestic animals or pets		
Never	1.00 (reference)	-
Sometimes	0.98 (0.83-1.16)	0.820
Almost every day	0.86 (0.73-1.00)	0.051
Attending religious activities		
Never	1.00 (reference)	-
Sometimes	0.89 (0.78-1.01)	0.076
Almost every day	0.90 (0.73-1.10)	0.311
Number of leisure activities		
0	1.00 (reference)	-
1	0.66 (0.60-0.73)	< 0.001
2	0.54 (0.49-0.61)	< 0.001
3	0.43 (0.37-0.49)	< 0.001
4+	0.42 (0.35-0.50)	< 0.001
P for trend		< 0.001

HR: hazard ratio; CI: confidence interval

[a] Adjusted for age and sex, residence, co-residence status, marital status, educational level, body mass index, smoking status, alcohol status, frequent fresh vegetable consumption, frequent fresh fruit consumption, physical exercise, hypertension, diabetes mellitus, respiratory disease, stroke, heart disease, depression symptoms, cognitive impairment, and participation in other leisure activities.
